# Neuromuscular adaptations in soleus and tibialis anterior muscles in persons with spinal cord injury

**DOI:** 10.1186/s12984-025-01794-7

**Published:** 2025-11-14

**Authors:** Asta Kizyte, Haocheng Zhang, Emelie Butler Forslund, Elena M. Gutierrez-Farewik, Ruoli Wang

**Affiliations:** 1 Department of Engineering Mechanics, KTH MoveAbility, KTH Royal Institute of Technology, Stockholm, Sweden; 2 Aleris Rehab Station, Research and Development Unit, Stockholm, Sweden; 3 Department of Neurobiology, Care sciences and Society, Karolinska Institutet, Stockholm, Sweden; 4 Department of Women’s and Children’s Health, Karolinska Institutet, Stockholm, Sweden; 5 Division of Paediatric Neurology, Department of Women’s and Children’s Health, Karolinska Institutet, Solna, Sweden

**Keywords:** Motor unit, Neural control, High-density EMG, Spinal cord injury, Neuromechanics

## Abstract

**Background:**

Spinal cord injury (SCI) can lead to various neurophysiological changes, altering the neural motor control strategies. The lower limb muscles are of high importance for locomotion; however, there exists a significant knowledge gap in neurophysiological changes following SCI in these muscles. This study aims to explore the neuromuscular adaptations in the soleus and tibialis anterior muscles in persons with incomplete SCI.

**Methods:**

Ankle joint torque, high-density electromyography (HD-EMG) and motor unit parameters of tibialis anterior and soleus were analyzed during repeated sub-maximal voluntary isometric contractions (20% and 50% of the maximal torque) and compared to those from a control cohort.

**Results:**

We observed muscle-dependent alterations in motor control between the SCI and control groups. Namely, SCI group required significantly higher normalized EMG amplitudes than the control group to achieve the same contraction levels. At 50% contraction level, compared to control group, the SCI group motor units were recruited at lower thresholds in both muscles and fired at lower rates in the tibialis anterior muscle. We observed no significant differences in intramuscular motor unit coherence or muscle co-contraction between the two groups.

**Conclusions:**

The observed combination of between-group differences in motor unit behavior may indicate that even in muscles of high-functioning individuals with incomplete SCI, is a shift towards larger motor units in both tibialis anterior and soleus muscles. These results contribute to knowledge of neurophysiological modifications in major ankle muscles following a SCI and provide deeper insights into neurophysiological changes that can be used complementary to clinical SCI evaluation.

## Introduction

Spinal cord injury (SCI) disrupts the afferent and efferent neuron pathways, resulting in a wide variety of motor and sensory impairments at or distal to the level of injury. Lower leg muscles, particularly ankle dorsi- and plantarflexors, crucial for daily bipedal activities, are often affected after SCI. Partial recovery may occur during the acute phase after SCI via a number of neurophysiological adaptations [1] that may persist during the sub-acute and chronic phases. A deeper understanding of these persistent changes, as well as the neuron recovery after the injury and altered neural control strategies, is necessary to facilitate rehabilitation and long-term post-injury management. However, knowledge of the persistent neurophysiological adaptations in major ankle dorsiflexor and plantarflexor muscles is still limited.

To address this gap, peripheral measures such as electromyography (EMG) offer a valuable window into the motor control system. Both time and frequency domain characteristics of EMG, as well as qualitative features, e.g., visible presence or absence of EMG signals, provide insight into neurophysiological changes and muscle control after a neurological injury, indirectly reflecting the behavior of motor units (MUs). For instance, EMG amplitude analysis during volitional movement has proven to be a useful indicator of muscle weakness [2], particularly during recovery in the acute phase after SCI. Increase EMG amplitude and muscle strength observed during this phase have been attributed to MU reinnervation via collateral sprouting of adjacent motor neuron axons [1, 3]. Moreover, a positive correlation between EMG amplitude and clinically assessed muscle strength has been reported after SCI [4].

Frequency-domain characteristics such as intramuscular coherence offer complementary insights into the common neural drive between two different areas of the same muscle and are associated with altered MU synaptic drive. Intramuscular coherence is typically analyzed in specific frequency bands, each thought to reflect different sources of neural input: the δ (< 5 Hz) and α (8–15 Hz) bands are primarily associated with subcortical and spinal contributions [5, 6], the β band (15–30 Hz) reflects cortical drive [7], and the γ band (30–60 Hz) is considered indicative of fast corticospinal or high-level motor control processes [8]. In δ band specifically, the MU activity is known to have a strong correlation with force generation [9]. In the tibialis anterior (TA), reduced intramuscular γ frequency band coherence has been observed during maximal isometric voluntary contraction after SCI [10] and has been reported to increase as muscle function improves during the subacute phase after SCI [11]. However, these studies used EMG – EMG coherence (i.e., coherence between two EMG signals), for which estimation is sensitive to electrode placement [12], and the influence of different EMG pre-processing pipelines is ambiguous [13, 14]. Such limitations raised concerns about validity of EMG – EMG intramuscular coherence as a measure of common neural drive. Alternatively, employing spiking MU activity instead of bipolar EMG has been suggested as a more robust and reliable method to estimate coherence [15].

While EMG parameters and coherence analysis provides some insight into motor control, a deeper understanding of neuromechanism requires examining MU behavior directly. Intramuscular EMG has been frequently used to measure MU activity in both non-disabled populations (e.g [16, 17]). , and in persons with neurological pathologies, including SCI [18–20]. Alternatively, these in vivo observations can be made with non-invasive surface high-density (HD) EMG and signal decomposition. Several surface HD-EMG decomposition algorithms have been proposed (e.g [21–23]). and validated [22, 24–26]. Using either of these techniques, MU behavior was reported to be altered in persons with SCI. Some studies suggest that fewer MUs remain under voluntary control after SCI [27, 28], complicating muscle control. Muscle force gradation of the thenar muscles, for instance, was shown to be altered in persons with SCI due to altered MU recruitment and de-recruitment thresholds and low MU firing rates [18], and changes in MU firing rates were found, varying among muscles and their relations to the level of injury. Wiegner et al. reported lower MU firing rates during sustained voluntary contraction in biceps brachii and TA muscles but higher in triceps brachii [29] in persons with SCI. Another factor contributing to the difficulty of controlling force in persons with SCI may be increased MU firing rate variability, reflected by the increased variability of time periods between the adjacent MU spikes (inter-spike intervals, ISI), as observed in biceps brachii of persons with SCI during fatiguing submaximal isometric contractions [19]. However, most above-mentioned studies relied on intramuscular EMG and primarily focused on the upper limb muscles. Further research is still needed to clarify how SCI affects the neural control in lower limb muscles that are critical for locomotion.

Based on the existing studies, we hypothesize that SCI is associated with altered neural drive to the ankle muscles, reflected in changes across both muscle-level EMG metrics (time and frequency domain) and MU-level characteristics, including recruitment, firing behavior, and synchronization. To explore this, we investigated motor control in individuals with incomplete SCI during controlled ankle contractions using surface HD-EMG decomposition. A comprehensive understanding of EMG and MU characteristics could provide valuable information on the force generation and modulation strategies allowing effective ambulation after the SCI.

This exploratory study therefore aimed to characterize in vivo SCI-related neurophysiological changes in major ankle plantarflexor and dorsiflexor muscles during sub-maximal voluntary isometric contractions in high-functioning individuals with incomplete paraplegia. Specifically, we analyzed whether muscle-level EMG parameters (time and frequency domains) and MU -level parameters differ between individuals with SCI and control cohort. In addition, ISI variability was compared with torque variability. Through a tailored experimental design integrating HD-EMG with diverse analytical approaches, we systematically evaluated muscle- and motor unit–level characteristics of the ankle muscles in individuals with SCI, offering a novel perspective on post-injury motor control.

## Methods

### Participants

Twenty participants (9 females, age 58.9 ± 10.9 years (mean ± standard deviation), height 163.9 ± 3.5 cm, weight 79.8 ± 18.2 kg) with sub-acute and chronic incomplete SCI (median 2.2, min 0.6, max 17 years after injury) and 17 control participants (10 females, age 53.1 ± 12.4 years, height 170.2 ± 8.4 cm, weight 69.5 ± 13.1 kg) were recruited. The participants with SCI were a subset of the participants recruited for the study by Butler Forslund et al. [30]. The inclusion criteria were selected to ensure that participants with SCI could perform the test procedure, but would have less than full plantarflexor strength, i.e. (i) muscle strength between 2 and 4 (inclusive) on the Daniels and Worthingham scale [31], and (ii) spasticity less than 4 on the Modified Ashworth Scale [32, 33]. Inclusion criteria for control participants were no known neurological disorders and no lower limb injuries in the past six months. All participants with SCI underwent a clinical evaluation including International Standards for Neurological Classification of Spinal Cord Injury [34] for the assessment of SCI injury level with regards to the motor and sensory function. All participants were classified as ASIA grade D, meaning that at least half of the key muscle functions below the neurological level of injury are preserved. The muscles are capable of active movement and full range of motion against gravity. Ankle plantarflexor muscle hyper-resistance (spasticity) was found in six participants, their scores ranging from 1 to 3 (where 0 is no increase in muscle tone, 5 – affected joint is rigid). In participants with motor function asymmetry, measurements were performed on the side with weaker muscles. In persons with no motor function asymmetry, measurements were performed on the side with poorer sensory function. In non-disabled participants, measurement was performed on a randomly selected side. The data collection was carried out at Promobilia MoveAbility Lab (Stockholm, Sweden). The study was approved by the Swedish Ethical Review Authority (2020–02311, 2020–07067, and 2022-00629-02). All participants signed an informed consent after receiving oral and written information.

### Data collection

The participants performed isometric plantar- and dorsiflexion while seated comfortably on a chair, with their knee flexed at 90° and the ankle fixed at 0° (neutral position). The measurement rig used a force dynamometer (NEG1, OT Bioelettronica) with a calibrated load cell (CCt transducer s.a.s. Turin, Italy) positioned under the forefoot to measure both tension and compression. HD-EMG electrode grids were placed over the muscle belly of TA (OT Bioelettronica, 13 × 5 electrodes with one corner electrode missing, inter-electrode distance 10 mm) and medial side of soleus (SOL) (OT Bioelettronica, 4 × 8 electrodes, inter-electrode distance 8 mm) muscles. Before electrode placement, the skin was shaved, cleaned with an alcohol wipe, and abraded with an abrasive paste. The maximum voluntary contraction (MVC) was determined from the maximum dynamometer force in two repetitions; each sustained for 3s, with verbal encouragement, separated by a one-minute break. Participants were then shown visual feedback of their applied ankle torque and asked to follow a repeating trapezoidal torque profile consisting of a 5s ascent (linear increase in torque), 4s plateau at 20% or 50% of their MVC, 5s descent (linear decrease), followed by 10s rest. Four repetitions at each contraction intensity constituted one trial and the order of trials was randomized. The protocol was motivated by the need to minimize fatigue in participants with SCI while capturing MU behavior across multiple MVC levels and recruitment slopes, ensuring representative MU activity under varied conditions for analysis. HD-EMG and ankle torques in the sagittal plane were recorded with a multichannel biological signal amplifier (Quattrocento, OT Bioelettronica) at a sampling rate of 2048 Hz and fixed amplification gain of 150 V/V.

### Data analysis

#### Ankle torque

Dynamometer readings were low pass filtered with a cut-off frequency of 10 Hz. Each trial’s torque data were divided into four repetitions and further segmented into ascent, plateau, and descent. The torque coefficient of variation (CoV_TRQ_) was calculated for the plateau segments to evaluate the variability of the sustained force at each MVC level.

#### EMG data

HD-EMG signals were band-pass filtered between 20 and 500 Hz using a second-order zero-phase Butterworth filter. The HD-EMG channels that were still noisy (i.e. excessive baseline noise relative to neighboring electrodes) after filtering or had no signals were manually removed from further processing. EMG amplitude was analyzed as normalized root mean square (NRMS_EMG_) by normalizing root mean square values of monopolar HD-EMG from each electrode in the grid to the root mean square value of the same electrode during the MVC. The mean NRMS_EMG_ of all electrodes and repetitions was then computed for each muscle and person. At each time point $\:t$, the mean NRMS_EMG_ of all the channels within each grid was used to calculate the co-contraction index (CCI) of antagonist muscle pair (TA and SOL) as:

1 $$\:\text{C}\text{C}\text{I}\left(t\right)\:=\:\frac{2{a}_{S}\left(t\right)}{\left({a}_{S}\right(t)+{a}_{L}(t\left)\right)},$$

where $\:{a}_{S}$ is the NRMS_EMG_ of the less active muscle and $\:{a}_{L}$ is the NRMS_EMG_ of the more active muscle [35]. The HD-EMG data of TA was used to analyze dorsiflexion contractions, the data for SOL was used to analyze plantarflexion contractions, and both were used to compute CCI.

In order to obtain MU spike trains, monopolar band-pass (20–500 Hz) filtered HD-EMG signals were decomposed using the convolutive blind source separation method [21, 36] implemented in DEMUSE software (version 6.0; University of Maribor, Slovenia). The MU spike trains identified by the algorithm were further visually inspected and manually edited by two well-trained investigators in accordance with recommendations by Del Vecchio et al. [37]. All MU spike trains with pulse-to-noise ratio above 30 [38] and less than 10 firing events in the time segment of interest (ascent, plateau, or descent) were excluded from further analysis. Timings between consecutive firing events, i.e. ISI, were computed. To remove physiologically unrealistic firings and periods of MU de-recruitment, only 33.3 ms < ISI < 200 ms were considered for further analysis [39, 40]. The coefficient of variation of the ISI (CoV_ISI_) during the plateau was computed for each MU as the ratio of mean and standard deviation of the ISI. MU discharge rates (the number of MU firings per second) were estimated during ascent, plateau, and descent as ratios of the sampling frequency and ISI. Recruitment and de-recruitment thresholds, defined as the mean torque during the first five firing events in torque ascent (MU recruitment) or the last five firing events in torque descent (MU de-recruitment), were computed for each MU. Finally, MU action potential (MUAP) waveforms were estimated with the spike-triggered averaging and the amplitude was calculated as the difference between the negative and positive MUAP peaks.

To estimate the MU synchronization in the frequency domain, the data combined from the plateaus of all four repetitions (resulting in 16s of data) was used to calculate the intramuscular coherence. Six randomly selected MUs from the MU pool were divided into two groups and combined into cumulative spike trains (CSTs) for each of the groups. To match these requirements, we could only use the data where at least 6 MUs continuously firing throughout the four repetitions were detected. The power spectral density for each CST was estimated using the Welch method [41], with 0.5s window size and 50% overlap before calculating intramuscular coherence $\:{C}_{xy}$ as:

2 $$\:{\left|{\text{C}}_{\text{x}\text{y}}\left({\updelta\:}\right)\right|}^{2}\:=\:\frac{{\left|{\text{f}}_{\text{x}\text{y}}\left({\updelta\:}\right)\right|}^{2}}{{\text{f}}_{\text{x}\text{x}}\left({\updelta\:}\right){\text{f}}_{\text{y}\text{y}}\left({\updelta\:}\right)},$$

where $\:{f}_{xy}$ is the cross power spectral densities between the two CSTs x and y, $\:{f}_{xx}$ and $\:{f}_{yy}$ are the power spectrum of x and y CSTs, respectively [42], $\:{\updelta\:}$ is the frequency at which the $\:{C}_{xy}$ is calculated. The process was repeated for up to 100 random permutations of MU groups. The integrals of $\:{C}_{xy}$ values were computed over the δ, $\:{\upalpha\:}$, $\:{\upbeta\:}$, and $\:{\upgamma\:}$ frequency bands for each permutation separately and the median value of all permutations was taken as the final coherence value.

In addition to coherence, the MU synchronization was addressed in the time domain by calculating the synchronization index of common input strength (CIS) [43]. The index was calculated for the same MU CST groups as the intramuscular coherence by (i) calculating the cross-correlogram of the two CSTs spanning 100 ms before and after the reference spike; (ii) establishing the baseline from the first 60 ms of the cross-correlogram output; (iii) identifying the peak using the cumulative sum method; and (iv) calculating the CIS as the identified peak count in the cross-correlogram divided by the length of the recording.

All signal processing and data analysis was performed with MATLAB R2022b software.

### Statistical analysis

The outcome parameters considered in this study include torque parameter CoV_TRQ_; EMG parameters: NRMS_EMG_ and CCI; and MU parameters: MU discharge rates, recruitment, and de-recruitment thresholds, CoV_ISI_, MUAP amplitude, CST coherence and synchronization index CIS. Statistical comparison was performed only between the control and SCI group. No comparisons were made between muscles (i.e., SOL and TA).

For parameters determined for each individual MU (i.e. MU discharge rates, recruitment, and de-recruitment thresholds, CoV_ISI_, and MUAP amplitude), statistical significance was assessed using robust linear mixed models implemented in the R function *rlmer()*. In these models, group was treated as fixed effects and subjects, number of identified MUs per subject, biological sex and age were treated as random effects. The p-values were then evaluated with the *modelsummary* library [44] and differences were considered statistically significant at *p* < 0.05.

For all other parameters (i.e. torque parameter CoV_TRQ_, EMG parameters, CST coherence and CIS), the medians of each parameter were computed at 20% and 50% MVC for the TA and SOL of each participant. Specifically, EMG parameters (NRMS_EMG_ and CCI), medians were computed from all HD-EMG channels and all movement repetitions at 20% or 50% MVC. When the assumption of normal distribution was violated (determined by the Shapiro-Wilk Test), statistical significance was determined by the Mann-Whitney U test (significance level *p* < 0.05). Otherwise, independent samples t-tests (*p* < 0.05) were used.

## Results

### Torque parameters

**Fig. 1:**
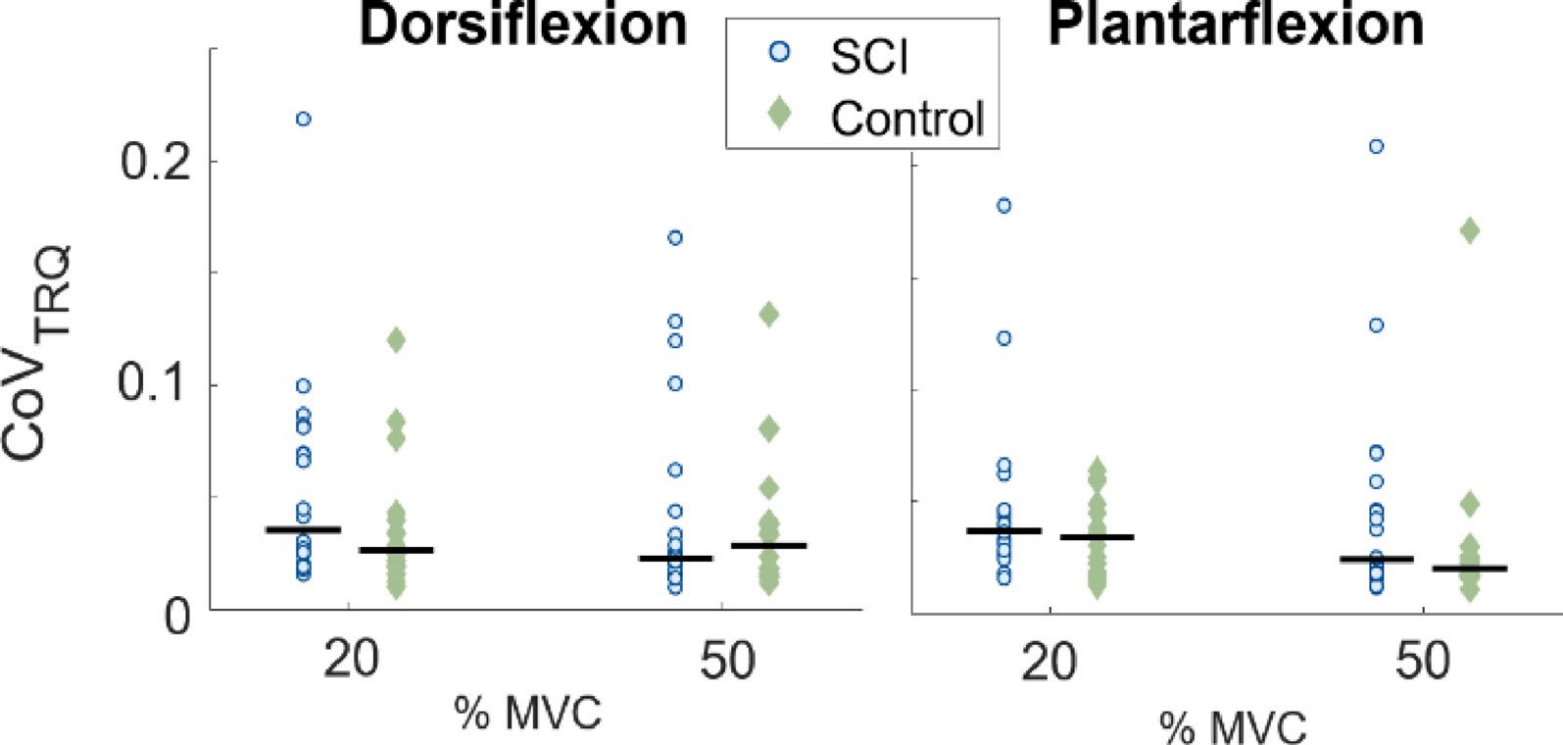
Coefficients of variation of torque CoV_TRQ_, quantifying the variation of torque during the dorsiflexion and plantarflexion plateau, shown for SCI and control groups at 20% and 50% MVC plateau. Each dot represents the median of one subject, and the black horizontal lines indicate the group median for the given MVC level. Higher CoV_TRQ_ reflects higher variation of measured torque

No statistically significant differences in mean CoV_TRQ_ between SCI and control groups were found (Fig. 1; dorsiflexion *p* = 0.220 at 20% MVC, *p* = 0.912 at 50% MVC; plantarflexion *p* = 0.2857 at 20% MVC, *p* = 0.236 at 50% MVC). However, a few outliers in the SCI group contribute to a higher CoV_TRQ_ variability in the SCI group compared to the control group during dorsiflexion (std. dev. SCI: 0.047 at 20% and 0.046 at 50% MVC; control: 0.038 at 20% and 50% MVC) and plantarflexion (SCI: 0.039 at 20% MVC, 0.047 at 50% MVC; control: 0.034 at 20% MVC, 0.033 at 50% MVC), indicating that several participants with SCI struggled maintaining constant torque.

### EMG parameters

**Fig. 2:**
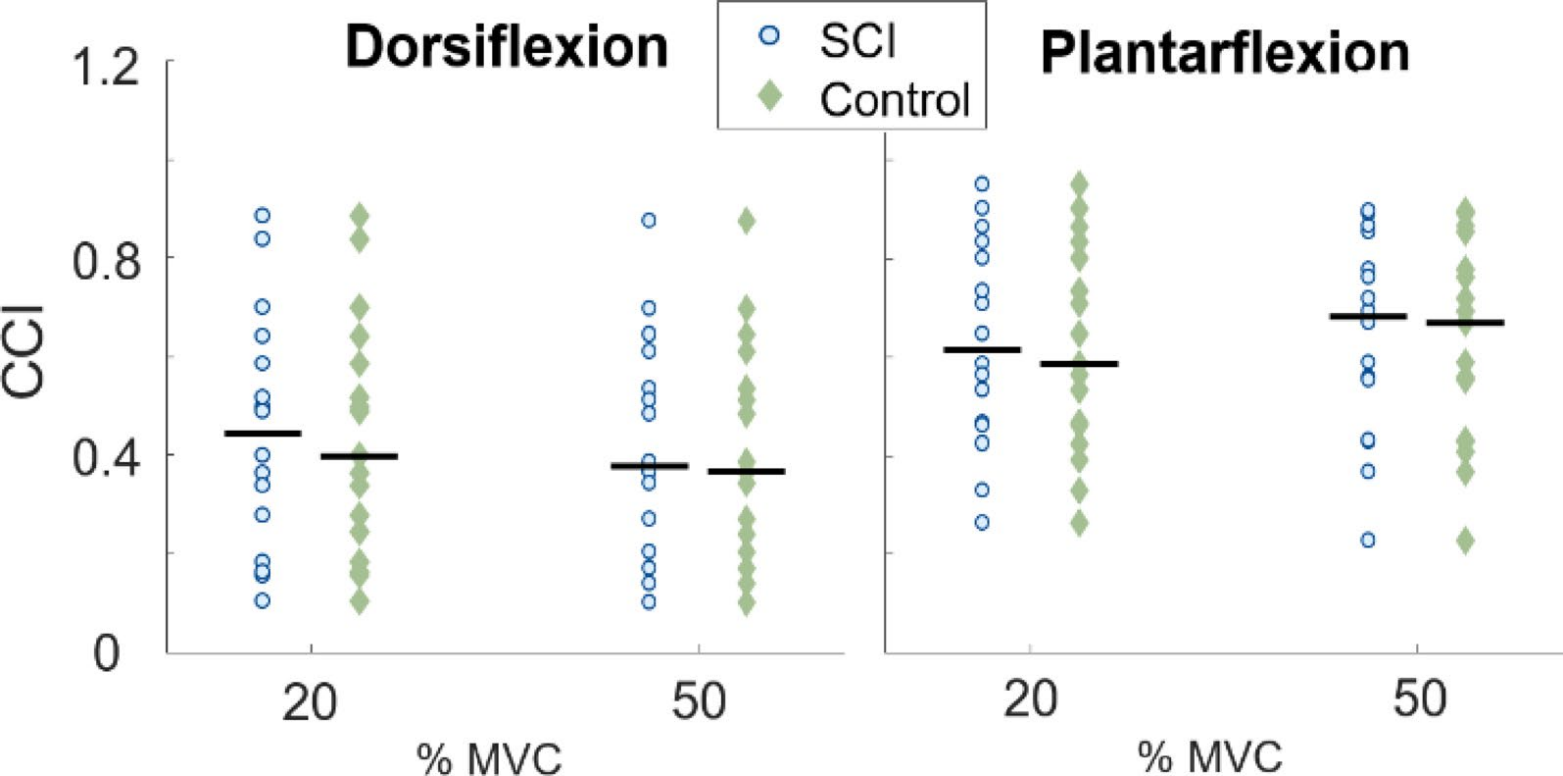
Co-contraction index CCI quantifying the co-contraction of soleus and tibialis anterior muscles during dorsiflexion and plantarflexion, shown for SCI and control groups at 20% and 50% MVC plateau. Each dot represents the median of one subject, and the black horizontal lines indicate the group median for the given MVC level. Higher CCI reflects stronger co-contraction

**Fig. 3:**
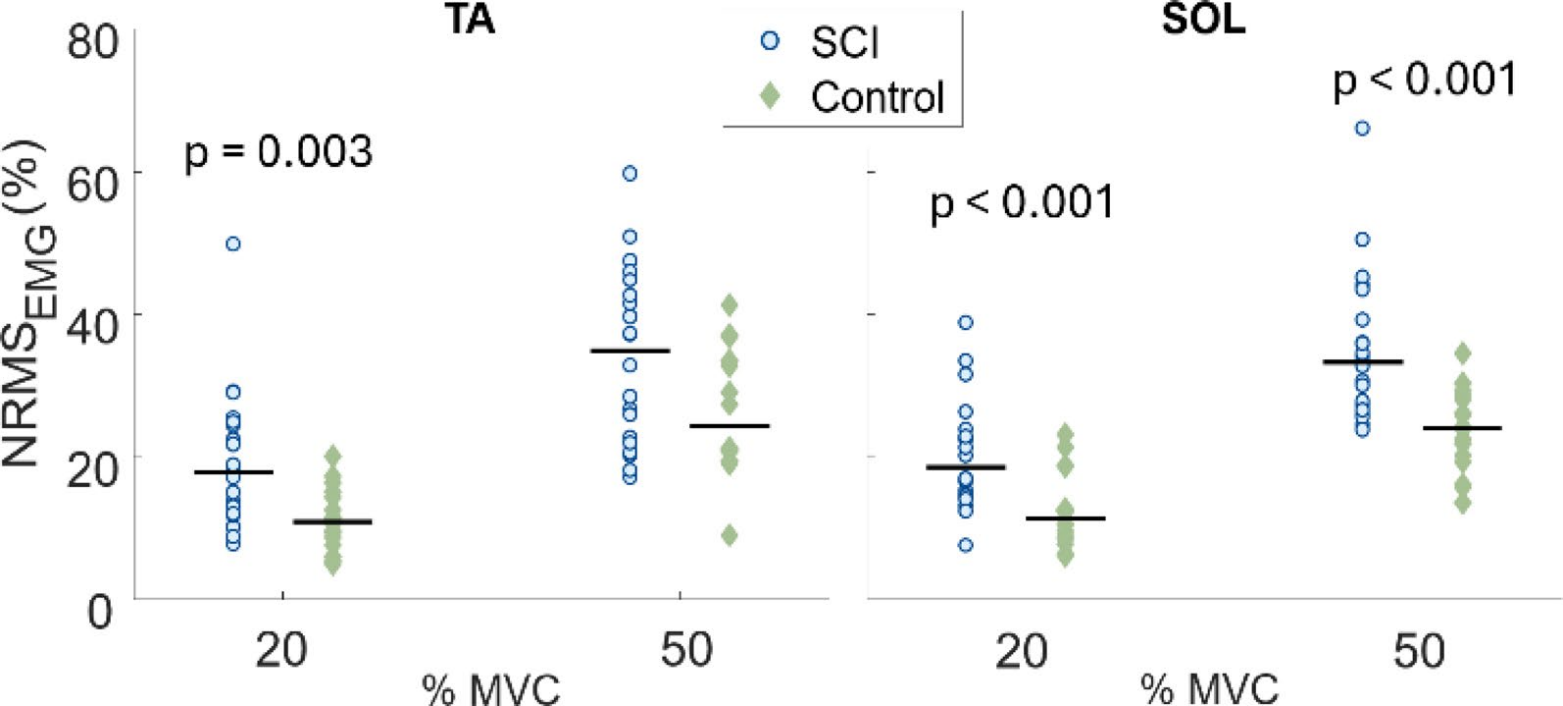
EMG amplitude of the tibialis anterior (TA) and soleus (SOL) muscles, normalized to peak EMG amplitude from the MVC trial, shown for the SCI and control groups at 20% and 50% MVC plateau. Each dot represents the median of one subject for the given muscle and MVC level, and the black horizontal lines indicate the group median for the given MVC level. Statistical difference between groups is indicated above each figure. NRMS_EMG_ reflects the percentage of maximum EMG contraction values needed to achieve the same relative torque level (MVC)

No significant differences in CCI between SCI and control group were found, (Fig. 2). Normalized EMG amplitude required to achieve 20% and 50% MVC levels was significantly higher in the SCI group than in the control group in all cases except in TA at 50% MVC (*p* = 0.062; Fig. 3). In the SCI group, the SOL was 55% and 51% higher than the control group at 20% and 50% MVC, respectively. Relative magnitudes of TA EMG were also higher in the SCI than in the control group (47% at 20% MVC and 43% at 50% MVC).

### MU parameters

Within subjects, typically a higher number of MUs were identified in TA than SOL (Appendix A), while between groups, a higher median number of MUs were identified in the SCI group than in the control cohort. In the SCI group, for each trial there were a median of 11 (9.5 in women, 12 in men; min 0, max 39) MUs identified in TA and 6 (4 in women, 7 in men; min 0, max 22) MUs identified in SOL per person. In the control group, for every trial there were a median of 7 (4 in women, 11 in men; min 0, max 33) MUs identified in TA and 2.5 (2 in women, 3 in men; min 0, max 19) in SOL per person.

**Fig. 4:**
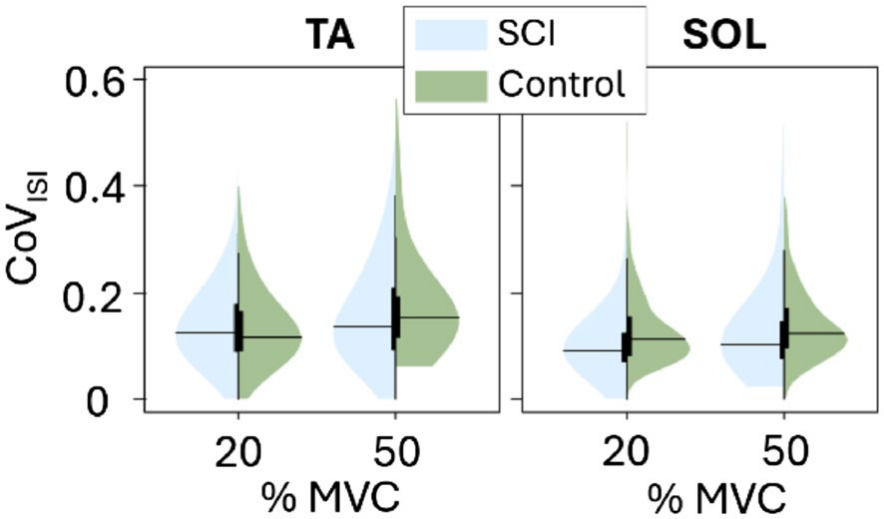
Coefficients of variation of inter-spike intervals CoV_ISI_ quantifying the variability of the motor unit firing pattern of tibialis anterior (TA) and soleus (SOL), shown for SCI and control groups at 20% and 50% MVC plateau. The black horizontal lines indicate the mean for the given group and MVC level. Higher CoV_ISI_ values represent more variable patterns

**Fig. 5:**
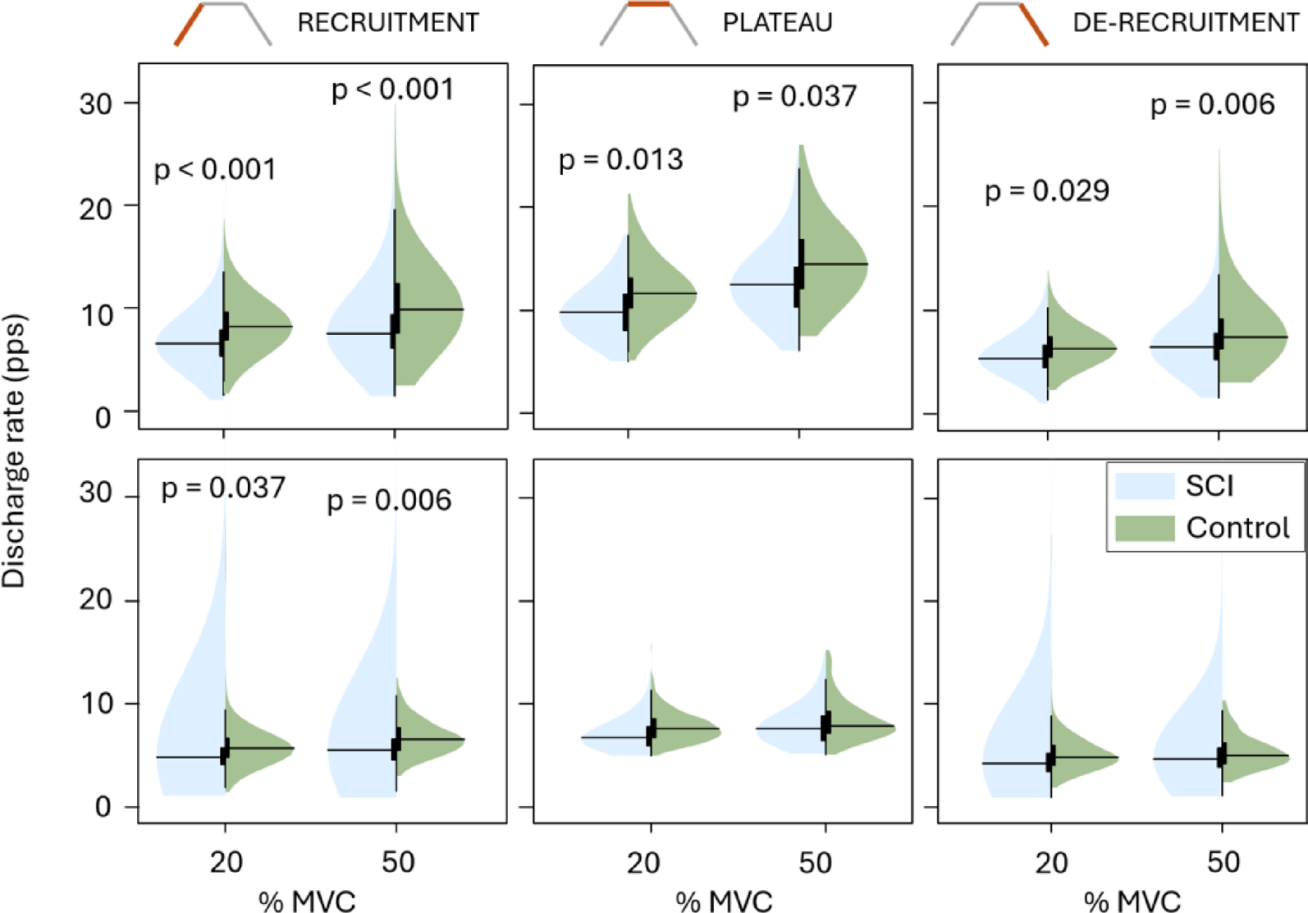
Tibialis anterior (top row) and soleus (bottom row) motor unit discharge rates (measured in pulses per second) of the SCI and control groups during motor unit recruitment, de-recruitment, and torque plateau shown for the trials plateauing at 20% and 50% MVC. The black horizontal lines indicate the mean for the given group and MVC level. Statistical difference between groups is indicated above each figure

ISI variability during the plateau was similar between groups (Fig. 4) both in TA (*p* = 0.997 at 20% MVC and *p* = 0.756 at 50% MVC) and in SOL (*p* = 0.702 at 20% MVC and *p* = 0.7907 at 50% MVC). Moreover, we found no significant differences between groups in SOL MU discharge rates (Fig. 5) except during recruitment. However, compared to the control group, the TA MU discharge rates were significantly lower in the SCI group at all MVC levels and movement phases.

**Fig. 6:**
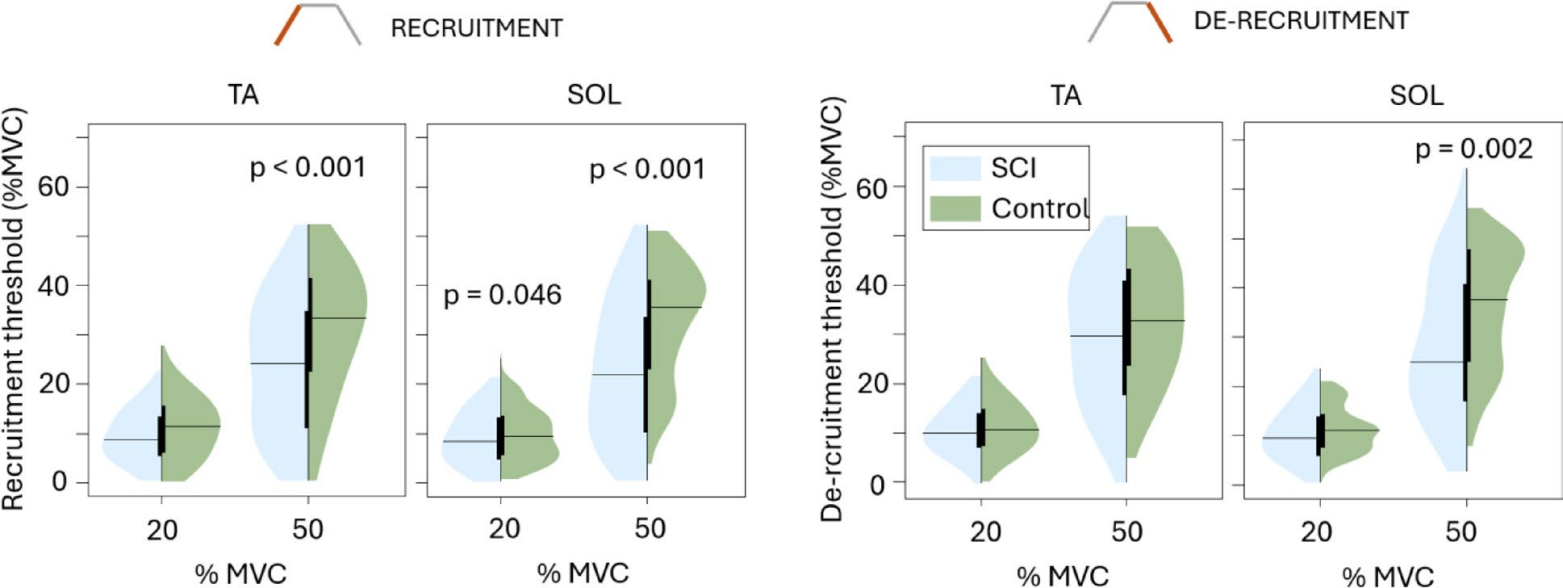
Tibialis anterior (TA) and soleus (SOL) motor unit recruitment and de-recruitment thresholds, reflecting the torque level at which the motor units start firing continuously, shown for SCI and control groups at 20% and 50% MVC plateau. The black horizontal lines indicate the mean for the given group and MVC level. Statistical difference between groups is indicated above each figure

The TA recruitment thresholds were significantly lower in the SCI group than in the control group, except recruitment at 20% MVC (*p* = 0.090, Fig. 6). The differences in mean de-recruitment thresholds were not significant (in TA *p* = 0.763 at 20% MVC, *p* = 0.143 at 50%; in SOL *p* = 0.208 at 20%); the only significant difference was in de-recruitment of SOL MUs at 50% MVC where SCI group mean was lower than control.

There were no significant differences in MUAP amplitudes both in TA (*p* = 0.114 at 20% MVC and *p* = 0.059 at 50% MVC) and SOL (*p* = 0.062 at 20% MVC and *p* = 0.316 at 50% MVC). Nonetheless, a trend was observed in which the SCI group exhibited higher mean MUAP amplitudes in TA compared to controls, whereas in SOL, the SCI group showed lower mean amplitudes relative to the control group.

**Fig. 7:**
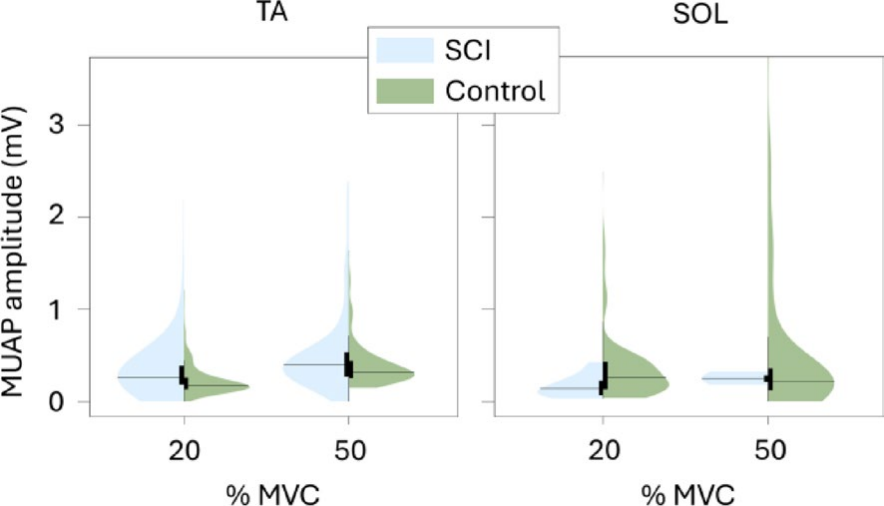
Tibialis anterior (TA) and soleus (SOL) motor unit action potential (MUAP) amplitudes estimated with spike-triggered averaging, shown for SCI and control groups at 20% and 50% MVC plateau. The black horizontal lines indicate the mean for the given group and MVC level. Statistical difference between groups is indicated above each figure

**Fig. 8:**
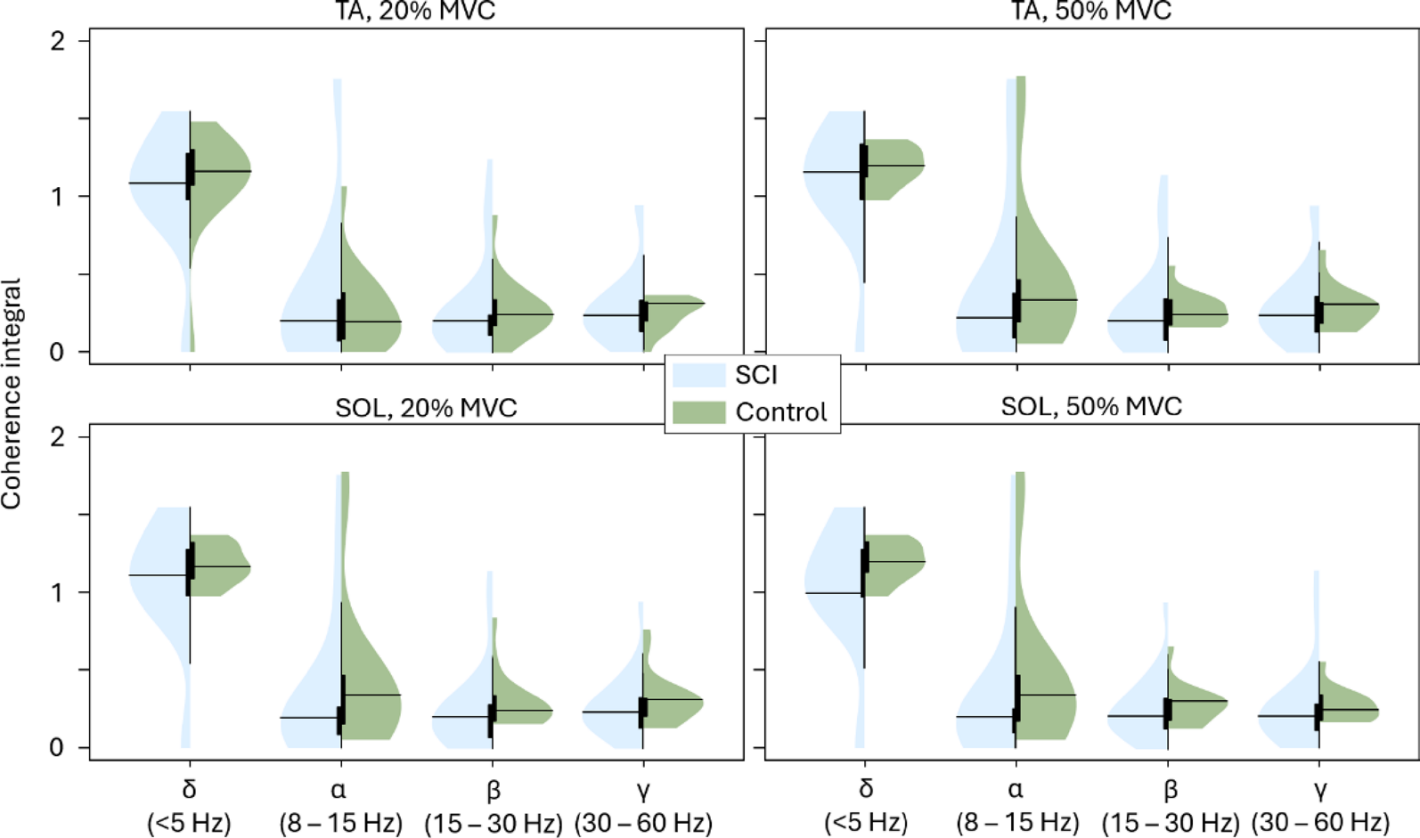
Tibialis anterior and soleus cumulative spike train coherence integral in δ, α, β, and γ frequency bands at 20% and 50% MVC, shown for SCI and control groups. The black horizontal lines indicate the mean for the given group and MVC level. Statistical difference between groups is indicated above each figure

**Fig. 9:**
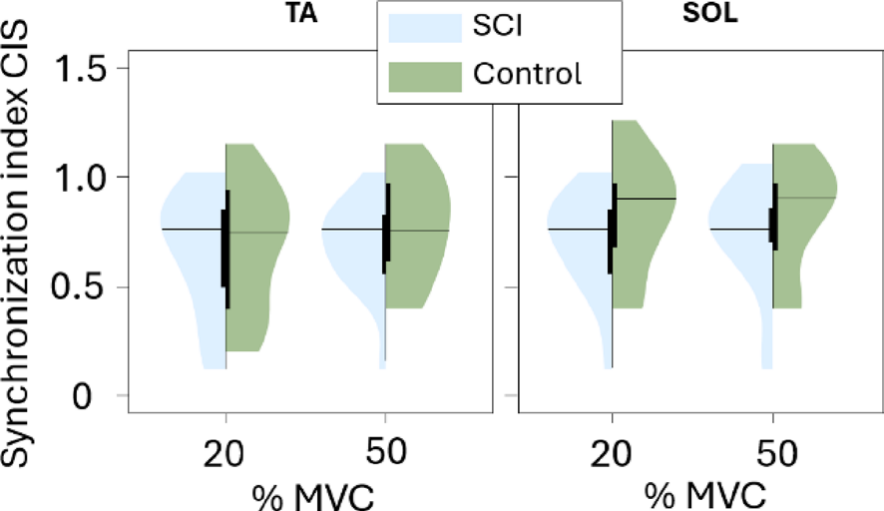
Synchronization index CIS for the cumulative spike trains of the Tibialis anterior (TA) and soleus (SOL) muscles at 20% and 50% MVC, shown for SCI and control groups. The black horizontal lines indicate the mean for the given group and MVC level. Higher CIS values reflect higher synchronization between the motor unit groups

Among different frequency bands, the intramuscular coherence was the highest in the δ band regardless of the group and contraction level. In TA, MU CST coherence was similar between groups across all frequency bands (Fig. 8). Similarly, SOL MU CST coherence was systematically lower in SCI group than the controls in all cases but none of the differences were statistically significant. The CIS index (Fig. 9) also showed no significant differences in the MU synchronization between the two groups in both TA (*p* = 0.155 at 20% MVC and *p* = 0.139 at 50% MVC) and SOL (*p* = 0.139 at 20% MVC and *p* = 0.139 at 50% MVC), although mean SOL MU synchronization was notably lower in SCI group compared to control.

## Discussion

To explore potential neurophysiological adaptations in TA and SOL muscles after SCI, we investigated EMG and MU properties of major ankle plantarflexor and dorsiflexor muscles at low and moderate intensity sub-maximal isometric contractions with HD-EMG. We found that participants with SCI required higher normalized EMG amplitudes to achieve the same level of normalized torque. Upon further analysis, we observed significant between-group differences in MU recruitment and de-recruitment thresholds, MUAP amplitudes, and TA MU discharge rates. However, no differences in MU synchronization were observed between the groups. These differences between the two participant groups highlight the altered isometric force modulation and generation strategies that may occur after an SCI and suggest a tendency to recruit larger MUs in the ankle dorsi-/plantarflexor muscles.

Altered isometric force generation strategies after SCI were evident in observed between-group differences in normalized EMG amplitude, coupled with lower SCI group MU firing rate. We found that participants with SCI needed significantly higher normalized EMG amplitudes to achieve the same levels of normalized torque as control participants. One possible factor contributing to this could be greater co-contraction, however, there was no significant between-group differences in co-contraction index observed, though the CCI varied greatly among individuals in the SCI group. Several other neural adaptations or their combinations could also possibly lead to a higher normalized EMG amplitude, including a greater number of recruited MUs, increased MU firing rate and/or synchronization, and increased MU size, wherein a neuron innervates more muscle fibers, resulting in a higher contribution from a single MU to the generated muscle force. We observed that, compared to control group, the SCI group’s SOL discharge rates were only significantly lower during the recruitment phase, whereas TA discharge rates were found to be significantly lower in all movement phases. These findings are also in line with Wiegner et al. [29]. Moreover, further intramuscular coherence analysis indicated a similar common drive within the muscle in the SCI group, suggesting that increased MU synergy is unlikely a motor control strategy to increase force production in persons with SCI. This observation is further confirmed by the CIS synchronization index showing similar MU synchronization in the time domain between the two groups. Considering all parameters in the EMG and MU level, it is most likely that the higher normalized EMG amplitudes observed in the SCI group could be achieved by recruiting more or larger MUs rather than by greater MU firing rates and synchronization. Although MUAP amplitudes were not significantly higher in the SCI group compared to the control group, this does not rule out the possibility of larger MUs being recruited in SCI group. It is important to note that while MUAP amplitude can provide an approximate indication of MU size, it is also influenced by factors such as the distance of the MU from the electrodes and the specific acquisition parameters of the EMG system [45, 46]. Previous studies have reported reduced counts and increased size of active MUs in thenar muscles in persons with SCI [28, 47] as measured with EMG during nerve stimulation and voluntary contractions. However, the abovementioned studies only investigated the thenar muscles, and EMG amplitude cannot directly indicate the size of the MUs. Thus, other explanations for this phenomenon, e.g., that more MUs are recruited to achieve the same torque, cannot be ruled out. Moreover, possible morphological changes associated with muscle atrophy following SCI [48] may also contribute to the reduced force production capacity.

Significantly lower mean MU recruitment and de-recruitment thresholds observed in the SCI group in both TA and SOL, indicating an altered isometric force modulation strategy, especially at higher force levels. In contrast to our findings, Zijdewind et al. reported that MUs of the thenar muscles were recruited up to a higher threshold in persons with SCI [18]. However, the MU recruitment range is narrower in the thenar muscle than in TA [49], likely due to differences in functional requirements of their motor control. Thenar muscles are adapted for high precision and dexterity tasks, while TA primarily focuses on force production. It is thus likely that the muscles would adapt differently to denervation associated with SCI. Additionally, increased discharge rate variability was found to be associated with increased isometric force variability in hand muscles [50]. We did not observe such a link between the CoV_ISI_ and CoV_TRQ_, nor did we observe significant differences of these parameters between groups.

We observed no significant differences in the MU synchronization between the two groups. Contrarily, a study by Bravo-Esteban et al. found lower intramuscular TA γ band coherence in participants with SCI during MVC [10], which implied that the neural recovery might not solely rely on corticospinal neuroplasticity, but also on changes in reticulospinal and propriospinal tracts. It is important to note, however, that there are several differences in the methodology between the two studies, most notably that the study by Bravo-Esteban et al. studied EMG – EMG coherence, while we studied CST – CST coherence. Although the trial duration in the current study was relatively short (16 s, compared to the recommended minimum of 20 s [37]) and may have reduced the reliability of the coherence estimation, the consistent observations from the time-domain analysis (CIS) support the robustness of our findings. Further analysis is needed to clarify whether the discrepancies between the findings could be attributed to factors of neurological or of methodological origin.

Analysis of EMG parameters has the advantage of easy implementation but at the cost of limited inference of the underlying neural mechanisms. MU decomposition allows direct non-invasive observation of MU parameters but is labor-intensive. The number of identified MUs may vary greatly between persons, muscles, and contraction conditions and the decomposition algorithm. We identified fewer MUs in SOL than in TA, which reduces the robustness of the SOL results. This, coupled with inherited large variability in the possible alterations caused by the SCI, makes it challenging to confidently interpret the results. Another limitation in this study is our focus on only isometric contractions; this is however the only reliable option for decomposition of surface HD-EMG measurements. More MU parameters, such as estimated MU size and number, could further enhance our understanding of neural adaptation mechanisms after SCI. Moreover, using methods complementary to EMG, such as electric or magnetic nerve stimulation, could provide further information, such as the twitch properties of the MUs [51] or estimated MU number and muscle membrane properties [52]; these warrant further study.

Through exploration of EMG and MU parameters, we observed several muscle-dependent adaptations in persons with SCI. Most notably, in persons with SCI, higher normalized EMG amplitudes were needed to achieve the same relative torques as in the control group. Not only were normalized EMG amplitudes higher at similar MVC levels, they were also achieved with similar (SOL) or lower (TA) MU discharge rates. Moreover, MUs were recruited at lower mean torques at moderate contraction level. All these observations indicate a possible shift towards larger MUs after a SCI in both TA and SOL muscles. A comprehensive HD-EMG based analysis at both muscle and MU levels complements conventional functional clinical assessments and provide additional valuable insight into neuromuscular adaptations occurring after SCI.

## Data Availability

The data supporting the findings of this study are available on request from the corresponding author, RW. The data was not made publicly available to protect the privacy of the research participants.

## Appendix A: number of decomposed MUs per subject

**Table:**

Group	Subject	%MVC	No. MUs in TA (repetitions 1 to 4)	No. MUs in SOL (repetitions 1 to 4)
SCI	S1	20	4, 4, 4, 4	2, 2, 2, 2
		50	1, 3, 1, 2	1, 2, 2, 3
	S2	20	11, 13, 14, 7	2, 2, 2, 2
		50	8, 3, 6, 4	1, 1, 1, 1
	S3	20	12, 14, 17, 14	9, 11, 8, 8
		50	0, 4, 1, 0	7, 6, 7, 8
	S4	20	1, 6, 4, 1	7, 6, 7, 4
		50	26, 26, 26, 26	6, 7, 6, 7
	S5	20	8, 9, 9, 10	0, 3, 2, 3
		50	8, 8, 6, 9	9, 9, 9, 9
	S6	20	29, 30, 30, 30	6, 8, 6, 7
		50	36, 37, 39, 37	7, 6, 7, 7
	S7	20	7, 8, 9, 8	5, 5, 5, 4
		50	2, 2, 3, 2	7, 6, 6, 6
	S8	20	11, 10, 12, 10	5, 3, 6, 6
		50	0, 0, 0, 0	4, 4, 4, 4
	S9	20	11, 11, 11, 11	3, 3, 1, 1
		50	16, 17, 17, 17	3, 3, 4, 4
	S10	20	1, 0, 1, 0	2, 4, 5, 6
		50	24, 24, 23, 23	8, 9, 3, 4
	S11	20	29, 29, 29, 25	11, 9, 9, 10
		50	31, 32, 31, 32	13, 14, 15, 13
	S12	20	5, 5, 4, 4	8, 8, 8, 8
		50	2, 5, 2, 2	10, 10, 9, 9
	S13	20	16, 15, 17, 17	2, 2, 3, 3
		50	14, 15, 15, 14	1, 1, 1, 1
	S14	20	18, 23, 23, 21	22, 21, 21, 21
		50	22, 18, 20, 23	15, 20, 18, 17
	S15	20	15, 14, 15, 15	9, 10, 10, 9
		50	13, 12, 6, 10	8, 8, 4, 7
	S16	20	4, 4, 4, 4	16, 16, 16, 16
		50	7, 7, 7, 7	14, 14, 15, 14
	S17	20	10, 13, 15, 0	4, 2, 3, 0
		50	10, 9, 8, 0	4, 2, 4, 0
	S18	20	21, 25, 24, 24	13, 13, 13, 15
		50	24, 27, 27, 23	7, 7, 5, 7
	S19	20	4, 4, 8, 10	7, 11, 8, 0
		50	20, 19, 22, 0	8, 12, 13, 0
	S20	20	11, 12, 10, 11	3, 6, 6, 5
		50	16, 14, 20, 18	6, 8, 7, 7
	C1	20	0, 1, 3, 2	4, 5, 7, 5
		50	21, 19, 24, 24	3, 5, 4, 3
	C2	20	0, 1, 0, 1	4, 2, 4, 4
		50	0, 0, 0, 0	2, 1, 1, 2
	C3	20	32, 33, 26, 30	10, 12, 11, 12
		50	15, 16, 17, 21	3, 4, 4, 4
	C4	20	8, 8, 7, 3	2, 3, 2, 2
		50	2, 4, 5, 1	2, 3, 2, 3
	C5	20	7, 6, 6, 6	1, 1, 1, 1
		50	3, 4, 2, 3	0, 0, 0, 1
	C6	20	1, 2, 1, 2	2, 2, 2, 2
		50	0, 0, 0, 0	0, 1, 1, 1
	C7	20	25, 23, 20, 23	8, 7, 6, 5
		50	19, 19, 21, 19	1, 3, 1, 1
	C8	20	4, 4, 3, 3	1, 1, 1, 1
		50	3, 2, 2, 3	0, 0, 0, 0
	C9	20	5, 6, 2, 3	1, 3, 1, 1
		50	0, 0, 1, 0	0, 1, 0, 0
	C10	20	17, 16, 16, 15	2, 1, 3, 3
		50	9, 11, 12, 12	0, 1, 1, 1
	C11	20	15, 16, 15, 15	5, 5, 5, 5
		50	11, 14, 15, 14	1, 2, 2, 2
Control	C12	20	23, 23, 23, 23	11, 12, 12, 12
		50	18, 18, 19, 19	6, 6, 5, 5
	C13	20	9, 9, 7, 10	3, 4, 3, 3
		50	9, 8, 9, 10	3, 3, 3, 4
	C14	20	4, 7, 6, 7	5, 7, 6, 5
		50	1, 1, 5, 1	11, 11, 11, 11
	C15	20	11, 11, 10, 12	19, 18, 19, 0
		50	0, 0, 0, 0	0, 0, 0, 0
	C16	20	3, 4, 4, 0	2, 2, 1, 2
		50	2, 1, 1, 2	0, 0, 0, 0
	C17	20	12, 12, 12, 12	3, 2, 3, 3
		50	13, 13, 13, 13	10, 10, 10, 10

## Appendix B: median EMG signal-tonoise ratio per subject

The signal-to-noise ratio values were calculated with OTBioLab + software from the recordings captured at the maximum voluntary contraction recordings before starting the dorsi-/plantarflexion movements. We present the median value for the electrodes placed on both tibialis anterior (TA) and soleus (SOL) electrode grids.

**Table:**

Group	Subject	Dorsiflexion		Plantarflexion	
		Tibialis anterior	Soleus	Tibialis anterior	Soleus
SCI	S1	37.21 dB	32.95 dB	23.89 dB	28.27 dB
	S2	26.37 dB	26.35 dB	24.40 dB	23.23 dB
	S3	22.18 dB	19.54 dB	21.26 dB	20.37 dB
	S4	19.19 dB	25.08 dB	10.11 dB	21.81 dB
	S5	24.34 dB	22.41 dB	17.68 dB	22.78 dB
	S6	28.73 dB	17.78 dB	20.85 dB	23.46 dB
	S7	22.77 dB	26.61 dB	10.94 dB	21.45 dB
	S8	50.86 dB	50.51 dB	22.42 dB	30.97 dB
	S9	38.63 dB	38.06 dB	50.11 dB	32.60 dB
	S10	31.89 dB	24.92 dB	17.12 dB	20.15 dB
	S11	27.02 dB	20.82 dB	20.70 dB	21.00 dB
	S12	40.26 dB	38.72 dB	27.09 dB	27.60 dB
	S13	29.83 dB	22.61 dB	26.44 dB	25.80 dB
	S14	28.25 dB	21.46 dB	32.91 dB	36.83 dB
	S15	31.83 dB	25.92 dB	30.78 dB	26.83 dB
	S16	32.12 dB	26.51 dB	25.45 dB	30.14 dB
	S17	43.45 dB	42.01 dB	38.16 dB	38.59 dB
	S18	34.52 dB	33.94 dB	30.28 dB	33.37 dB
	S19	19.55 dB	16.13 dB	16.69 dB	21.44 dB
	S20	28.70 dB	22.43 dB	16.55 dB	20.65 dB
Control	C1	33.95 dB	23.98 dB	22.14 dB	20.01 dB
	C2	32.10 dB	27.46 dB	21.81 dB	22.68 dB
	C3	32.29 dB	30.31 dB	26.12 dB	28.22 dB
	C4	36.27 dB	31.56 dB	15.63 dB	21.70 dB
	C5	30.48 dB	24.89 dB	23.68 dB	27.46 dB
	C6	34.93 dB	35.67 dB	23.22 dB	31.00 dB
	C7	36.73 dB	35.86 dB	29.22 dB	31.55 dB
	C8	34.22 dB	26.77 dB	22.28 dB	23.35 dB
	C9	24.94 dB	27.08 dB	11.36 dB	22.82 dB
	C10	39.28 dB	50.43 dB	34.40 dB	56.57 dB
	C11	36.21 dB	33.03 dB	34.44 dB	35.60 dB
	C12	41.66 dB	37.24 dB	34.03 dB	34.84 dB
	C13	31.02 dB	33.90 dB	21.13 dB	23.99 dB
	C14	33.68 dB	31.48 dB	29.99 dB	32.12 dB
	C15	42.38 dB	39.11 dB	34.64 dB	35.01 dB
	C16	40.49 dB	29.01 dB	21.70 dB	16.05 dB
	C17	31.97 dB	31.98 dB	19.56 dB	22.93 dB

## Abbreviations

CCI: Co-contraction index
CIS: Common input strength
CoV_TRQ_: Coefficient of variation of torque
CoV_ISI_: Coefficient of variation of inter-spike intervals
CST: Cumulative spike train
EMG: Electromyography
HD-EMG: High-density EMG
MU: Motor unit
MVC: Maximum voluntary contraction
NRMS_EMG_: Normalized root mean squared error of the EMG
SCI: Spinal cord injury
SOL: Soleus
TA: Tibialis anterior
